# The effect of pain neuroscience education on chronic postsurgical pain after total knee arthroplasty: a randomized controlled trial

**DOI:** 10.2340/17453674.2024.41346

**Published:** 2024-08-28

**Authors:** Dominique C BAAS, Johanna C van AALDEREN-WICHERS, Tjeerd H VAN DER GOOT, Ronald J VERHAGEN

**Affiliations:** 1 Department of Orthopedics, Tergooi MC Hilversum; 2 Department of Medical Psychology, Tergooi MC Hilversum; 3 Altrecht, Psychosomatic Medicine, Zeist, the Netherlands

## Abstract

**Background and purpose:**

Chronic postsurgical pain after total knee arthroplasty (TKA) is frequent and may be reduced by pain neuroscience education (PNE), teaching people about pain from a neurobiological perspective. This study investigated primarily the effectiveness of 2 individual sessions of PNE versus usual care on pain levels 3 months postoperatively in patients undergoing TKA. Secondary outcomes were physical functioning, stiffness, health-related quality of life, pain catastrophizing, attention to pain, and levels of anxiety and depression.

**Methods:**

A prospective single-center, parallel-group randomized controlled trial was undertaken including patients aged 18 years or older scheduled for primary TKA. 68 patients were randomly assigned to PNE or usual care. The primary outcome was the Western Ontario and McMaster Universities Osteoarthritis Index (WOMAC) pain score 3 months postoperatively. Outcomes were measured preoperatively, at 2 weeks (acute phase), and at 3 and 12 months postoperatively.

**Results:**

We found no statistically significant difference (0.4 points; 95% confidence interval [CI] –1.7 to 2.4) in WOMAC pain scores 3 months after TKA between the PNE and control group. We found a statistically significant difference between the 2 groups for attention to pain at 3 months in favor of PNE (P = 0.02).

**Conclusion:**

This RCT showed that PNE was not superior to usual care in terms of reducing pain at 3 months after TKA. Attention to pain, as a secondary outcome, was significantly lower in the PNE group compared with usual care. Other secondary outcome measures showed no significant differences.

Even though many people experience alleviation of knee pain after total knee arthroplasty (TKA) [1], 10–34% report chronic pain after primary TKA [2,3]. Given this considerable number, it is important to investigate effective methods in reducing postsurgical knee pain. Pain neuroscience education (PNE) refers to educational interventions aimed at teaching individuals about the biological and physiological processes involved in the experience of pain [4]. If patients understand that pain results not only from tissue damage, they can adjust their beliefs, attitudes, behaviors, treatment, and lifestyle choices, which might positively influence their pain experience [5].

Systematic reviews showed that PNE is effective in reducing chronic musculoskeletal pain with a small to moderate effect [6-8]. Only a few studies focused on chronic postoperative pain in TKA patients. However, no consensus was reached on the effect of PNE on pain, fear of movement, and pain catastrophizing [9-11].

Providing PNE sessions individually may be important to facilitate support of patients’ own conceptualization of pain, in contrast with group sessions. We expected PNE to be more powerful when delivered before and after surgery, to support pain experiences of patients to reduce risk of chronic pain following TKA.

Our primary aim was to compare the effectiveness of 2 individual PNE sessions with usual care on pain levels 3 months after surgery in patients undergoing TKA. Further, the secondary aim was to evaluate the effects of PNE on physical functioning, stiffness, health-related quality of life, pain catastrophizing, attention to pain, and levels of anxiety and depression.

## Methods

### Participants

A prospective, single-center, 2-arm, parallel group, assessor blinded, randomized controlled trial was conducted at Tergooi MC Hilversum, the Netherlands. Patients 18 years or older with a primary diagnosis of osteoarthritis, according to the American College of Rheumatology classification criteria [12], scheduled for an elective unilateral TKA were recruited between August 2019 and September 2021. Table 1 presents the criteria for inclusion and exclusion. Patients were informed about the procedures and gave written informed consent prior to participation. Radiographic disease severity (Kellgren–Lawrence 0–4 grading scale) [13] was evaluated for each participant.

**Table 1:** Inclusion and exclusion criteria

**Inclusion criteria** Be able to understand and speak Dutch language. Patient is willing and able to complete scheduled study procedures and follow-up evaluations.
**Exclusion criteria** Scheduled for revision arthroplasty. Diagnosis of inflammatory arthritis (i.e., rheumatoid arthritis, psoriatic arthritis, systemic lupus erythematosus, ankylosing spondylitis). Scheduled for TKA because of a fracture, malignancy, or an infection. Patient is currently participating in any other surgical intervention studies or pain man-agement studies. Previous total knee arthroplasty or any other lower limb surgery within the past 6 months. Cognitive impairment (diagnosis of MCI or dementia).

The study is reported according to the CONSORT statement. It was registered at the Netherlands Trial Register (Trial Registration NL67769.041.18)

### Control group

Patients in the control group received usual care. This consisted of an appointment with an orthopedic consultant approximately 2–4 weeks before surgery in which patients received an instruction booklet outlining the TKA procedure. All patients were advised to request physiotherapy at a local practice. Stitches were removed approximately 2 weeks after surgery. Furthermore, all patients had control visits with the orthopedic surgeon 8 weeks and 12 months after surgery. Medication for pain control was routinely prescribed.

### Intervention group

The intervention group received care as usual with the addition of 2 face-to-face PNE sessions. The first 45-minute educational session took place approximately 1–3 weeks before surgery and the second 30-minute session coincided with the stitches removal appointment approximately 2 weeks after surgery. Both PNE sessions took place at the physiotherapy department of Tergooi MC Hilversum. The PNE sessions were delivered by the same physiotherapist, specifically trained on the PNE protocol by a specialized PNE trainer. The content of the sessions was tailored to the patient’s experiences before surgery, based on a collection of 9 formulated target concepts (Table 2). The physiotherapists kept track of the targets discussed with the patient and observed each other on a regular basis to make sure that they delivered the intervention in the same way. 3 to 4 times a year, the physiotherapists were supervised by the specialized PNE trainer.

**Table 2:** Target concepts PNE. Targets were tailored to the needs of the patient and delivered by the same physiotherapist trained by a specialized PNE trainer

Learning about pain can help the individual. Pain is normal, personal, and always real. Pain relies on context. Pain depends on the balance of perceived danger and safety. Pain is one of many protective outputs of the body. Danger and threat can increase sensitivity of protecting systems. The human body is bioplastic. Active treatment strategies promote recovery. Pain and tissue damage rarely relate.

### Outcome measures and follow-up

***Primary outcome.*** The primary outcome measure was pain at 3 months after TKA surgery with a focus on clinically relevant reductions in pain. The Dutch version of the Western Ontario and McMaster Universities Arthritis Index (WOMAC-DV), subscale pain, was used as pain-specific instrument. The scale consists of 5 items; overall scores run from 0–20. Items are rated using 1 of 5 responses (0 = none, 1 = mild, 2 = moderate, 3 = severe, 4 = extreme). The WOMAC-DV is a self-report questionnaire, to measure the pain associated with the knee in the last 48 hours. The psychometric properties are adequate: the internal consistency (α = 0.99) and test–retest reliability (ICC = 0.77) of the Dutch version are high [14]. By using the minimum clinically important difference (MCID) as a threshold (2.4 points on a 0–20 scale), we defined whether the differences in improvements in pain within each group were clinically meaningful [15].

***Secondary outcomes.*** Secondary outcomes included physical functioning, stiffness, health-related quality of life, pain catastrophizing, attention to pain, and levels of anxiety and depression. We used the subscales stiffness and physical functioning of the WOMAC-DV. The scale stiffness consisted of 2 items, scores ranging from 0–8 (stiffness) and physical functioning of 17 items, with scores ranging from 0–68. Higher scores mean more complaints. The “Pain Catastrophizing Scale Dutch Version” (PCS-DV) was used to address feelings and thoughts related to pain [6]. Total scores range from 0–52. The “Pain Vigilance and Awareness Questionnaire” (PVAQ) measures awareness, consciousness, vigilance, and observation of pain [7]. The scores range from 0–80. Presence and severity of anxiety and depression are determined with the “Hospital Anxiety and Depression Scale” (HADS) [8]. The HADS consisted of 2 scales and is applicable for detecting changes over time. The sum score for both scales is a minimum of 0 and a maximum of 21. General health-related quality of life was assessed with the dimension well-being of the RAND-36 [9]. This dimension consists of 3 subscales: mental health, vitality, and pain. The pain subscale of the RAND-36 refers to all possible pain complaints, not only knee-related complaints. Raw scale scores are transformed to a 100-point scale. Higher scores mean better quality of life. All outcomes were measured at baseline, 4–8 weeks preoperatively (T0), at 2 weeks (T1), 3 months (T2), and 12 months after surgery (T3).

### Deviations from protocol due to COVID-19

Due to COVID-19, regular orthopedic care was phased out for a period in 2020 and 2021, planned surgeries were postponed and deviations to standard procedure occurred, e.g., assessments out of range and PNE offered by telephone instead of face to face.

### Sample size

We used the statistical power analysis program G*Power (https://www.psychologie.hhu.de/arbeitsgruppen/allgemeine-psychologie-und-arbeitspsychologie/gpower). The difference in pain-related function on the 20-point WOMAC pain scale was used to calculate the needed study sample. Based on former studies we used an MCID for the subscale pain of 2.4 [15] between the 2 groups, with a medium effect size of 0.5 (using the Cohen effect size specification). To detect superiority of the PNE intervention over care as usual, we estimated a sample size of 30 participants per treatment group with a statistical power of 0.90 and a 2-sided significance (alpha) level of 0.05. Considering a potential loss to follow-up of up to 20%, the plan was to recruit 76 patients.

### Randomization and blinding

Variable block randomization, with stratification at patient level on sex and Kellgren–Lawrence grade, was performed in a 1:1 ratio using the validated web-based system Castor EDC (2019). Considerable effort was made to avoid observer bias through separation of roles and blinding of trial staff. Orthopedic surgeons, the orthopedic consultant, and researchers were blinded to the type of treatment.

### Statistics

Descriptive statistics were used to describe the baseline characteristics of individuals in each group. Data was tested for normality using the Shapiro–Wilk test. Data is described by mean (standard deviation [SD]) and comparisons between study groups were performed using Student’s t-test for continuous variables, and by the chi-square for categorical variables. In the analysis the intention-to-treat principle was used including all randomized participants. A statistically significant difference between 2 sets of comparable data was defined as P < 0.05.

A sensitivity analysis related to multiple imputation was conducted to assess the robustness of the primary results.

Data from the outcome measures was analyzed with a repeated-measures ANOVA with treatment (PNE, usual care) as the between-subject factor, and time (baseline, 2 weeks after surgery, 3 months after surgery, and 12 months after surgery) as the within-subject factor and presented with 95% confidence intervals (CI). All statistical analyses were performed using IBM SPSS Statistics 28.0.1 (IBM Corp, Armonk, NY, USA).

### Ethics, registration, funding, and disclosures

All patients gave written informed consent, and the study was approved by the Medical Ethics Committee of the University Medical Center Utrecht (NL67769.041.18) and conducted in accordance with the Declaration of Helsinki. Tergooi MC Hilversum has no role in the study, other than supporting and stimulating scientific research. The authors report no competing interests. Complete disclosure of interest forms according to ICMJE are available on the article page, doi: 10.2340/17453674.2024.41346

## Results

### Patient flow

From August 2019 to September 2021, 151 patients were screened for eligibility. 74 patients were willing to participate and 68 were included in the final analysis (Figure).

**Figure:**
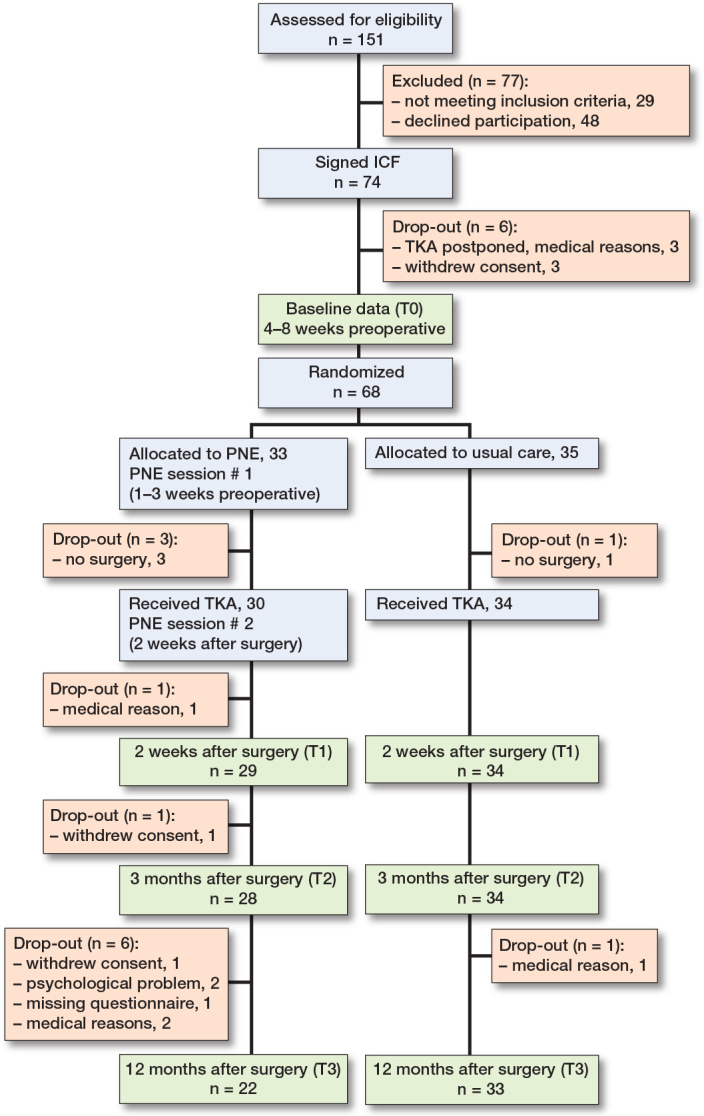
CONSORT flowchart of the trial and required assessments. ICF = informed consent form; PNE = Pain Neuroscience Education.

Of 68 patients randomized, 45 were female and the mean age was 68 years. The mode of the Kellgren–Lawrence grading scale was 4 and mean duration of knee pain was 66 months (Table 3). The primary outcome data was normally distributed, and the 2 groups had almost similar demographic and clinical characteristics at baseline (Table 3), except for the secondary outcome PVAQ (P = 0.03). The PNE group showed significantly higher baseline PVAQ measures.

**Table 3:** Baseline demographics and characteristics. Values are mean (SD) unless otherwise specified

Factor	PNE n = 33	Usual care n = 35
Age	68 (8)	69 (9)
Female sex, n	22	23
Body mass index	27 (4)	28 (4)
Months of pain	76 (103)	57 (98)
Kellgren–Lawrence grade, n		
0	0	0
1	0	0
2	1	1
3	7	6
4	25	28
Pain medication in the past week, n		
No analgesic use	21	21
Paracetamol	16	17
NSAIDs (e.g., ibuprofen, diclofenac)	8	5
Weak opioids (e.g., tramadol)	3	0
Strong opioids (e.g., oxycodone)	1	0
Primary outcome		
WOMAC-DV pain	8.7 (4.1)	8.5 (3.2)
Secondary outcomes		
WOMAC-DV stiffness	3.9 (2.2)	3.7 (2.3)
WOMAC-DV physical functioning	27.3 (15.2)	25.5 (12.9)
PCS-DV	10.6 (11.2)	6.7 (8.8)
PVAQ	34.8 (17.1)	26.2 (14.9)
HADS	6.1 (7.1)	6.0 (5.1)
RAND-36 mental health	85.9 (17.9)	86.6 (13.1)
RAND-36 vitality	70.3 (21.4)	69.9 (15.8)
RAND-36 pain	52.1 (25.5)	50.5 (22.8)

Abbreviations: PNE, Pain Neuroscience Education; WOMAC-DV, Western Ontario and McMaster Universities Arthritis Index Dutch Version; PCS-DV, Pain Catastrophizing Scale Dutch Version; PVAQ, Pain Vigilance Attention Questionnaire; HADS, Hospital Anxiety and Depression Scale; RAND-36, RAND-36-item Health Survey.

Missing values were considered to be missing completely at random, observed in 4 cases, < 1% of the total number of variables. Sensitivity analysis of the primary outcome measure, using multiple imputation methods, showed similar results.

### Deviations from protocol due to COVID-19

In 13 patients the baseline assessment did not take place within the set range. In 6 patients there was a delay in 1 or both sessions of PNE. Finally, in 6 patients, 1 or both PNE sessions took place by telephone, instead of face to face.

### Outcome measures

***Primary outcome.*** We found no between-group difference (mean difference of 0.4 points; CI –1.7 to 2.4) in the primary outcome measure WOMAC pain 3 months after TKA between the PNE and usual care group. The mean WOMAC pain score 3 months postoperatively was 4.2 (CI 2.5–5.9) for the PNE group and 3.8 (CI 2.5–5.1) for the usual care group. Both groups showed improvement in WOMAC pain scores from baseline to 3 months. The PNE group reduced their WOMAC pain score by a mean of 4.7 (CI 2.7–6.8) and the usual care group by a mean of 4.6 (CI 3.1–6.1) (Table 4).

**Table 4:** Primary and secondary outcomes at 2 weeks, and 3 and 12 months after surgery. Values are count or mean (CI)

Factor	PNE	n	Usual care	n	Mean difference between groups
Primary outcome					
WOMAC-DV pain					
2 weeks	5.4 (4.1 to 6.7)	28	4.7 (3.5 to 5.9)	34	0.7 (–1.0 to 2.4)
3 months	4.2 (2.5 to 5.9)	28	3.8 (2.5 to 5.1)	34	0.4 (–1.7 to 2.4)
12 months	0.9 (–0.2 to 2.0)	22	1.1 (0.4 to 1.8)	33	–0.2 (–1.4 to 1.1)
Secondary outcomes					
WOMAC-DV stiffness					
2 weeks	3.7 (3.2 to 4.2)	28	3.1 (2.5 to 3.6)	34	0.6 (–0.2 to 1.3)
3 months	2.7 (2.1 to 3.4)	28	2.3 (1.7 to 2.9)	34	0.4 (–0.5 to 1.3)
12 months	1.2 (0.6 to 1.7)	22	1.3 (0.8 to 1.9)	33	–0.2 (–1.0 to 0.7)
WOMAC-DV physical functioning					
2 weeks	18.0 (13.9 to 22.0)	28	17.2 (13.6 to 20.8)	34	0.8 (–4.5 to 6.1)
3 months	11.9 (7.9 to 15.8)	28	10.7 (7.2 to 14.2)	34	1.2 (–4.0 to 6.3)
12 months	3.6 (0.4 to 6.8)	22	4.7 (1.8 to 7.7)	33	–1.1 (–5.5 to 3.3)
RAND-36 mental health					
2 weeks	86.4 (79.6 to 93.2)	28	87.6 (83.7 to 91.6)	34	–1.2 (–8.6 to 6.2)
3 months	88.9 (84.2 to 93.5)	28	90.8 (85.9 to 95.6)	34	–1.9 (–8.6 to 4.8)
12 months	92.7 (87.4 to 98.0)	22	92.5 (89.2 to 95.7)	33	0.2 (–5.5 to 6.0)
RAND-36 vitality					
2 weeks	48.4 (42.7 to 54.1)	28	48.8 (43.7 to 54.0)	34	–0.4 (–7.9 to 7.1)
3 months	53.0 (47.9 to 58.1)	28	53.1 (48.7 to 57.5)	34	–0.1 (–6.6 to 6.5)
12 months	85.7 (77.5 to 93.8)	22	83.0 (78.3 to 87.8)	33	2.7 (–6.0 to 11.3)
RAND-36 pain					
2 weeks	59.3 (50.8 to 67.9)	28	52.9 (43.0 to 62.9)	34	6.4 (–6.8 to 19.5)
3 months	76.0 (67.1 to 84.9)	28	72.8 (66.8 to 78.7)	34	3.2 (–7.0 to 13.4)
12 months	90.2 (84.1 to 96.3)	22	88.1 (82.3 to 94.0)	33	2.0 (–6.5 to 10.6)
PCS-DV					
2 weeks	5.4 (3.0 to 7.8)	28	5.2 (2.7 to 7.7)	34	0.3 (–3.2 to 3.7)
3 months	3.2 (1.1 to 5.4)	28	3.0 (1.7 to 4.3)	34	0.2 (–2.2 to 2.6)
12 months	1.1 (–0.4 to 2.5)	22	2.3 (0.6 to 4.0)	33	–1.2 (–3.6 to 1.1)
PVAQ					
2 weeks	29.4 (23.4 to 35.4)	28	28.5 (24.0 to 32.9)	34	1.0 (–6.2 to 8.1)
3 months **^a^**	23.6 (17.1 to 30.2)	28	24.6 (20.8 to 28.4)	34	–0.9 (–8.4 to 6.5)
12 months	21.5 (15.6 to 27.4)	22	23.3 (18.3 to 28.4)	33	–1.9 (–9.5 to 5.8)
HADS					
2 weeks	4.7 (2.2 to 7.2)	28	4.4 (2.8 to 5.9)	34	0.3 (–2.5 to 3.1)
3 months	3.2 (2.0 to 4.4)	28	3.3 (2.3 to 4.3)	34	–0.1 (–1.6 to 1.4)
12 months	2.5 (0.6 to 4.3)	22	3.1 (2.0 to 4.3)	33	–0.7 (–2.7 to 1.3)

For abbreviations, see Table 3.

a P value is 0.02 from repeated measures within-subjects differences at 3 months after surgery, with time points as a categorical variable.

***Secondary outcomes.*** The between-group difference in WOMAC pain at 12 months postoperatively was a mean of 0.2 (CI –1.4 to 1.1), which was not statistically significant. We found lower attention to pain (PVAQ) scores in the PNE group compared with the usual care group 3 months after surgery, namely 23.6 (CI 17.1–30.2) vs 24.6 (CI 20.8–28.4) (Table 4). At 3 months, the PVAQ scores were reduced by a mean of 11.7 points (CI 5.5–17.9) in the PNE group vs 2.2 (CI –3.1 to 7.5) in the usual care group (P = 0.02). All other secondary outcomes at 3 months and 12 months after surgery showed no significant differences between the PNE and the usual care group. However, for several secondary outcomes, such as WOMAC-DV stiffness, WOMAC-DV physical functioning, PCS-DV, PVAQ, and HADS, a significant improvement was seen over time after TKA for both groups (Table 4).

## Discussion

The primary aim of this study was to compare the effectiveness of 2 individual PNE sessions in addition to usual care on chronic postsurgical pain in patients undergoing TKA.

This study failed to show superiority of PNE compared with usual care on WOMAC pain scores at 3 months. We did find significantly lower attention to pain (PVAQ) scores in the PNE group compared with the usual care group 3 months after surgery, but no additive effect of PNE for all other secondary outcomes. Yet, in both groups, WOMAC pain scores showed a clinically relevant improvement from baseline to 3 months. Furthermore, both groups showed improved scores for psychosocial variables such as catastrophizing, attention to pain, anxiety and depression, stiffness, and limitations in physical functioning over time, indicating that patients benefit from TKA.

In contrast to our hypothesis, the mean difference of 0.4 points in WOMAC pain scores 3 months after TKA between the 2 groups was not statistically significant. However, the CI includes the MCID, which supports the idea that the intervention might be beneficial for some patients, but that the size of the sample in this study was too small, which affected the CI. Despite some studies describing additional PNE as effective in the reduction of pain [6,20], Watson and coworkers [7] are cautious about offering PNE as a stand-alone intervention targeting pain and physical function, based on their findings in mixed-methods systematic review and meta-analysis.

There may be several explanations for the current findings. First, when interpreting the results, it is important to consider the consequences of the COVID-19 pandemic on our study population. COVID-19 has led to a significant delay in new patient influx, and deviations in the delivery of PNE, which might have influenced our results. By excluding patients who deviated from the protocol, the effect of PNE on the outcome measure became stronger. Watson and coworkers [7] showed that it is important for PNE sessions that patients are heard and understood, to recognize their understanding and beliefs regarding pain, promoting their readiness to engage with PNE. Lepri and co-workers [6] concluded in their systematic review that PNE is most effective when delivered in one-to-one oral sessions. It is possible that the protocol deviations worked counterproductively in the process of reconceptualization of the pain experience of patients. We decided to retain the study patients with protocol deviations, given the size of the groups, and the reliability of the analyzes.

Second, based on existing literature, it is expected that 10–34% of patients will experience moderate to severe chronic postsurgical pain [2]. These findings are not reflected in our study, as at 3 and 12 months after surgery a significant reduction of pain is seen in all participants, with mean pain scores lower than mild postsurgical pain. At 3 months postoperatively 19% of the patients report moderate to severe knee pain and at 12 months only 6%. However, nearly half of the patients approached to participate did not want to participate in the study, with an overall dropout of 26%. This was more than the estimated 20%. Frequently heard reasons are that patients find the questionnaires and possible intervention a hassle, in addition to the complaints and concerns they already experience in their lives. The current study group may therefore not be representative of TKA patients, which is an important consideration, as this could be the group for whom PNE is ideally suited. A systematic review by Lewis et al. [21] shows that depression, pain catastrophizing, preoperative pain, and the presence of other pain areas besides knee pain are significant predictors of postsurgical pain after TKA. It is plausible that the patients, who did not want to participate in the study or dropped out because of complaints and worry, score higher on pain, but also on psychological variables like pain catastrophizing, attention to pain, and anxiety and depression. Based on previous research [22-24] we hypothesize that PNE might be more effective for patients with high levels of these psychological variables. The systematic review of Siddal and coworkers [20] argued that the positive effect of PNE in combination with exercise may be related to the mediation of kinesiophobia and pain catastrophizing. The RCT by Birch et al. [24] investigated the effect of cognitive behavioral education in patients before TKA and included patients with a PCS score > 22. No previous research is known in which PNE is offered to a subset of patients after screening on a specific trait. Our current findings suggest that attention to pain is an interesting variable.

### Strengths

Our PNE sessions were based on a flexible protocol, consisting of 9 formulated target concepts. Based on the patient’s attitudes and beliefs, target concepts were chosen tailored to the patient, in line with Nijs’s recommendations [25]. Physiotherapists captured the specific targets delivered to each patient, which is useful for further research.

### Limitations

There are some limitations. First, PNE is delivered by physiotherapists, specifically trained on the PNE protocol but relatively inexperienced. There was no independent verification of the quality of the PNE delivered. Second, we have no information concerning patients who declined participation. Third, in this study usual care was chosen as the control group. In randomized controlled trials it is recommended to compare with an active control group. Otherwise, it is not possible to distinguish whether the effect of PNE on pain after TKA is due to the PNE session or due to professional attention during the period of surgery.

### Conclusion

The results of this RCT showed that PNE was not superior to usual care in terms of reducing pain at 3 months after TKA. Attention to pain, as a secondary outcome, was significantly lower in the PNE group compared with usual care. Other secondary outcome measures show no significant differences at any measurement points.

In perspective, future research is warranted to explore the effect of PNE by including patients with psychosocial comorbidities to optimize representation of patients with chronic postsurgical pain and improve quality of life and reduce physical complaints and healthcare consumption.

## Notes

DB and HvA: study setup, data collection, data-analysis, drafting of manuscript, revision of manuscript. TvdG: data collection, revision of manuscript. RV: original idea, interpretation of data, revision of manuscript. All the authors contributed to the content of the manuscript and approved the final version.

Handling co-editors: Li Felländer-Tsai and Philippe Wagner

*Acta* thanks Karin Bernhoff and Siri Heijbel for help with peer review of this manuscript
